# Electrical performance of PEDOT:PSS-based textile electrodes for wearable ECG monitoring: a comparative study

**DOI:** 10.1186/s12938-018-0469-5

**Published:** 2018-04-02

**Authors:** Reinel Castrillón, Jairo J. Pérez, Henry Andrade-Caicedo

**Affiliations:** 1 Mobile Computation and Ubiquituos Research Group GIMU, Universidad Católica de Oriente, Sector 3 Cra 46-40 B-50, Rionegro, Colombia; 2 Centro de Bioingeniería, Facultad de Ingeniería Eléctrica y Electrónica, Universidad Pontificia Bolivariana, Circular 1 #70-01, Medellin, 050031 Colombia

**Keywords:** Textile electrodes, PEDOT:PSS, Electric characterization, Contact impedance, ECG electrodes

## Abstract

**Background:**

Wearable textile electrodes for the detection of biopotentials are a promising tool for the monitoring and early diagnosis of chronic diseases. We present a comparative study of the electrical characteristics of four textile electrodes manufactured from common fabrics treated with a conductive polymer, a commercial fabric, and disposable Ag/AgCl electrodes. These characteristics will allow identifying the performance of the materials when used as ECG electrodes. The electrodes were subjected to different electrical tests, and complemented with conductivity calculations and microscopic images to determine their feasibility in the detection of ECG signals.

**Methods:**

We evaluated four electrical characteristics: contact impedance, electrode polarization, noise, and long-term performance. We analyzed PEDOT:PSS treated fabrics based on cotton, cotton–polyester, lycra and polyester; also a commercial fabric made of silver-plated nylon Shielde® Med-Tex P130, and commercial Ag/AgCl electrodes. We calculated conductivity from the surface resistance and, analyzed their surface at a microscopic level. Rwizard was used in the statistical analysis.

**Results:**

The results showed that textile electrodes treated with PEDOT:PSS are suitable for the detection of ECG signals. The error detecting features of the ECG signal was lower than 2% and the electrodes kept working properly after 36 h of continuous use. Even though the contact impedance and the polarization level in textile electrodes were greater than in commercial electrodes, these parameters did not affect the acquisition of the ECG signals. Fabrics conductivity calculations were consistent to the contact impedance.

## Background

The imminent population growth is a major concern for public health systems worldwide. In most countries, the capacity in hospitals result insufficient to opportunely treat patients. Traditional medicine is reactive rather than preventive based on late responses to, in many cases, predictable conditions. Furthermore, this system is deficient in covering the persistent demand, especially from cardiovascular patients, who require a continuous and frequent monitoring. According to the World Health Organization (WHO), cardiovascular diseases are listed as the globally leading cause of death with about 17.5 million deaths in 2012, accounting for 31% of deaths worldwide. Consequently, early diagnosis of these diseases becomes an essential means for their prevention and treatment [1].

Electrocardiography (ECG) is one of the most popular techniques in clinical practice [2]. Technological advances have permitted to include it in the daily life of patients. Modern biomedical systems allow the incorporation of high-performance ambulatory monitoring devices in commonly used elements such as clothing. These elements are known as wearable systems and belong to a strategic trend of technological devices that seek the improvement of the health care promotion. They have enabled continuous wearable monitoring of several physiological signals at a low cost, easily manufacturability and comfort.

A growing interest in alternative electrodes referred to as textile electrodes has been reported from different research groups [3]. The performance of textile electrodes has been evaluated in biological signals such as respiration and ECG monitoring and compared with commercial ECG-electrodes [4]. However, these textile electrodes have limitations on noise reduction, polarization, durability and long-term performance, that need to be overcome.

Although ECG monitoring systems traditionally depend on the utilization of Ag/AgCl disposable electrodes, textile electrodes offer an alternative means to register electrical cardiac readings over time, yielding equivalent diagnostic information. Ag/AgCl electrodes are suitable for short periods of time. Afterward, they will become uncomfortable due to the use of adhesives to enhance a firm attachment to the skin. They require the use of electrolytic gels that evaporates after few hours [5]. Additionally, they could eventually generate harmful skin reactions [6]. These problems make the conventional Ag/AgCl electrode unsuitable for routine and long-term ECG measurements.

Ag/AgCl electrodes have been extensively studied and tested, to the extent that the association for the advancement of medical instrumentation (AAMI) and the National Institute of American Standard (ANSI) have proposed the standard “Disposable ECG Electrodes—ANSI/AAMI EC12:2000/(R)2010” containing the performance requirements and test methods for disposable electrodes used in electrocardiography. Nevertheless, there is no similar standard for electro—conductive textile electrodes [7]. This fact leads the researchers to propose the most relevant and resourceful strategies in the characterization of textile electrodes.

Dry electrodes enable long-term monitoring that becomes relevant for specific health conditions, such as chronic diseases, fitness, and self-care. Current efforts aim to the development of ambulatory monitoring systems based on electrodes assembled from textile materials as cotton, polyester, lycra, and silver-plated nylon. Fabrics made of those materials are commonly utilized in wearable systems, since they are treated with compounds, such as electroactive polymers, carbon structures and metal substrates [8], that allow their electrical conduction and hence the detection of biological potentials. Poly(3,4-ethylenedioxythiophene)-poly(styrene sulfonate) or in simplified form, PEDOT:PSS [9, 10] are used to improve ionic conductivity, which reduces the effects of contact impedance in ECG signal acquisition.

Most of the works reported in the literature are comparative studies between new textile electrodes and commercial reference electrodes. In addition, many of them focus on testing contact impedance and noise. Pani et al. [11] treated woven cotton and polyester fabrics with highly conductive PEDOT:PSS solutions and introduced the effects on the conductivity and their affinity when using a different second dopant. Conductivity was reported for cotton = 424 mS/cm and polyeste = 575 mS/cm and compared with commercial Ag/AgCl 3M electrodes in human ECG recordings. The results showed that the conductivity can be improved by both improving the quality of the treatment of the textiles with conductive polymers and by carefully designing the electrode, i.e. the distribution of the surface, the thickness, snap fastener and conductive yarn. They indicated that there is a decrease in the mismatch in the electrochemical impedance of the skin-electrode interface when using PEDOT: PSS, therefore, conductive gels can be avoided. In this work, we studied the electrical performance of five fabrics from the point of view of polarization, long-term performance, contact impedance and noise.

Each electrode was evaluated based on four key features: contact impedance [12–16], polarization [17–19], noise level [7, 14, 20, 21] and long-term performance [6, 14, 17, 22].

## Methods

Samples of the textile materials used in this study were kindly provided by the Department of Electrical and Electronic Engineering, University of Cagliari, Cagliari, Italy. Fabrication process is described by Panni et al. [11]. Briefly, textile electrodes were made by treating conventional fabrics with a conductive solution of PEDOT:PSS dispersion Clevios PH 500 (Heraeus Clevios—Germany), second dopant was glycerol 33%. Woven fabrics were used, and immersed for at least 48 h at room temperature in the polymer solution. Fabrics were then taken out from the solution and drained off to remove the solution in excess. Samples were annealed, for both water and dopants to evaporate in order to avoid deterioration of the fabric mechanical properties. The conductive fabrics used in this study were: cotton, cotton–polyester (65% cotton, 35% polyester), lycra, polyester; additionally, we included a commercial silver-plated nylon fabric Shieldex®Med-Tex P130 (Statex—Germany) [23].

**Fig. 1:**
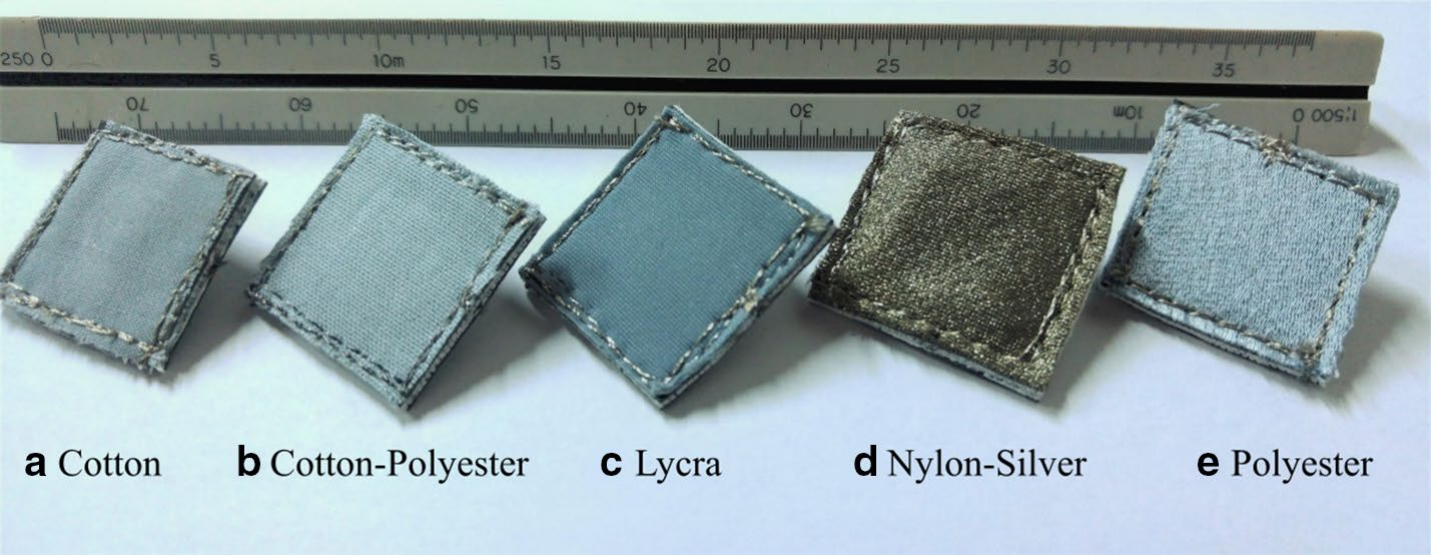
Physical appearance of the electrodes used in the research. Each electrode was constructed from 2 cm × 2 cm pieces of fabric sewn to a non-conductive support using silver–nylon conducting yarns. The commercial Ag/AgCl electrode is used as the standard of comparison in each of the measurements

Figure 1 shows five electrodes manufactured following the process described by Pani et al. [11]. The fabrics were cut into pieces of 20 mm × 20 mm, which were sewn to a non-conductive synthetic leather with silver-coated yarn to obtain greater rigidity. The size of the electrodes, which is acceptable for ECG monitoring, was chosen to ensure reproducibility of the customized fabrication process. The use of a layer of rigid synthetic leather allowed to improve the contact between the electrode and the skin ensuring a uniform pressure, which is especially beneficial in the case of textile electrodes.

**Fig. 2:**
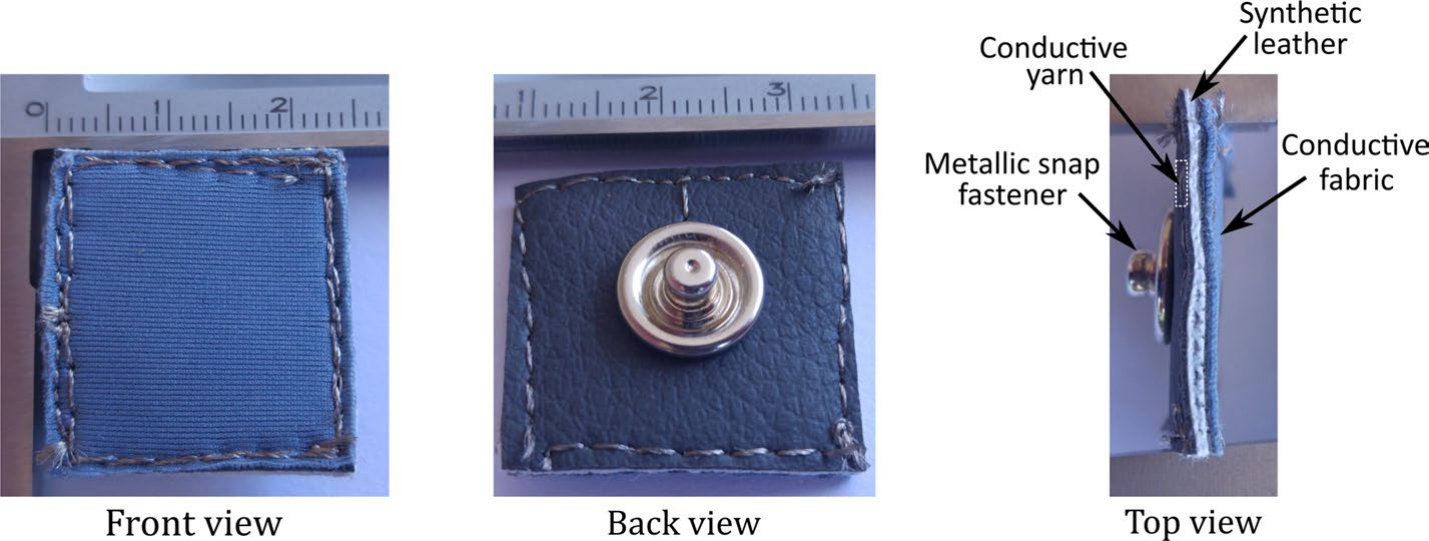
Closer view of the final aspect of the textile electrode. Front, back and top view of a textile electrode made of lycra. On the right side, it is possible to discern the elements of the electrode: conductive fabric, synthetic leather, conductive yarn, and metallic snap fastener

Finally, we fixed a metallic snap fastener to the synthetic leather and interfaced them with the same conductive yarn. In such a way, the snap fastener remained at the rear of the electrode without getting in touch with the skin. Figure 2 shows a closer view of the final aspect of the electrode, its structure, and components. The snap fastener was used to connect the electrodes to the ECG leads. In the experiments, we utilized Ag/AgCl disposable electrodes ref 2228 (3M, Germany) as the reference electrode on the ECG recording arrangement. This work focuses on electrode-skin interactions, other evaluation tests to characterize the physical properties of the electrodes were not conducted.

Pani et al. [11] reported the values of conductivity for cotton and polyester. In this work, we calculated the conductivity for cotton–polyester and lycra as the product between thickness and surface resistance, considering the fabrics as thin films with uniform surfaces, commonly reported as two-dimensional entities. Med-Tex P130 conductivity was not calculated as per its plated surface, it is intended to obtain silver ionic release for wound care, skin disorders, skin irritations, burn victims, not for uniformed conductivity.

**Fig. 3:**
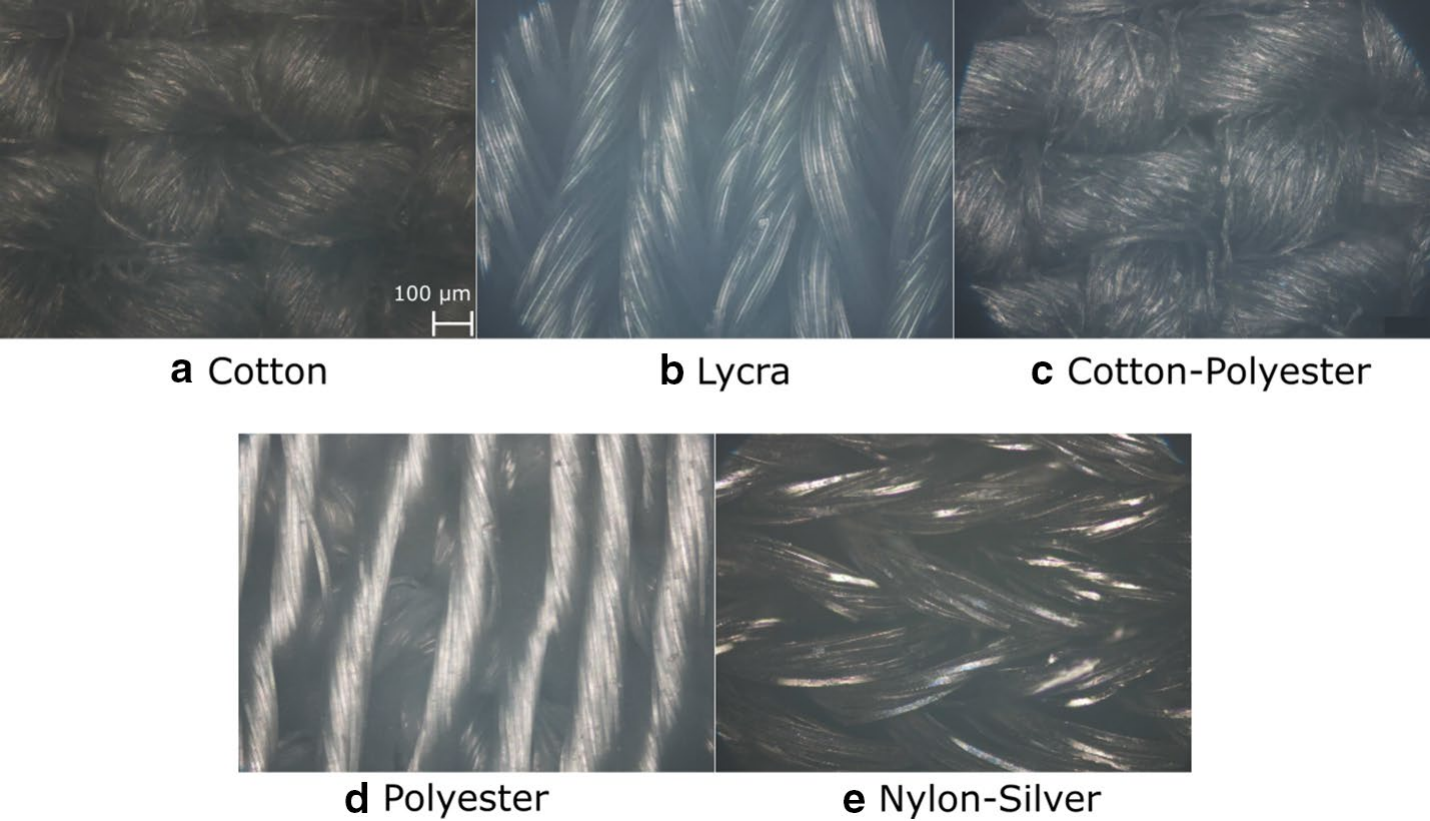
Optical micrographs of the fabrics used in the study. An upright microscope with a 10× objective was used. The scale bar corresponds to 100 µm

Figure 3 shows optical micrographs of each type of fabric where is possible to appreciate the different types of weave. Optical micrographs were acquired using a 10× objective and an upright microscope (Eclipse Ci; Nikon).

Our test protocol was previously approved by the Committee of Health Research Ethics of Universidad Pontificia Bolivariana (Colombia), located therein in the document R.R. N 80 17-12-2008. Data were obtained from 8 healthy, slim build individuals between the ages of 18 and 30, four for each test (two men and two women). Our interest lied in the number of repeated measurements of each type of electrode rather than in a large number of individuals. Even though the evaluations were carried out with four participants, the noise was measured in eight individuals: four individuals chosen originally for the noise test and other four resulting from the first long-term performance measurements since both tests followed the same protocol. We set the experiments in an in-paralleled electrode configuration. We replaced the disposable electrodes at every test to avoid adding a new variable into the experiments.

The volunteers were informed about the protocol to which they would be subjected. They were asked to be at rest for a period of 30 min before the test to homogenize their body conditions. Then, the area to be measured was shaved and cleaned with alcohol to improve the adhesion of the electrodes to the skin. An elastic waistband was used to attach the textile electrodes to the skin. Neither adhesives nor electrolytic gel were used.

We used an R language based platform known as Rwizard [24] to perform all the statistical analysis.

The main electronic equipment that we used in the study was:

- A virtual instrument, composed by a two channels USB oscilloscope and a function generator, Handyscope HS5 (Sneek, The Netherlands).
- A device to measure low voltages, currents, and power, Cassy Lab (LD DIDACTIC GmbH, Hürth, Germany).
- A switching circuit which is driven by a microcontroller to measure the power terminals at different points of the circuit.
- An acquisition card based on an EVM ADS1298 device, low-power, 24-bit, simultaneously sampling, eight-channel front-end for ECG and EEG applications (Texas instrument, Texas, EEUU).
- A laptop to set up the electronic systems and to record the data.

Measurement strategies are described in the following sections:

### Contact impedance measurements

Contact impedance refers to the impedance at the skin-electrode interface. This test intends to quantify the property of the electrode-skin contact to oppose time-varying electric current produced by the material under test. We selected single and double dispersion Cole impedance models of first and second order to represent this parameter [25, 26]. We set a variable AC source at 5 V peak to peak ($5~V_{pp}$) to sweep in a range of 0.1 Hz–10 kHz. Although the spectral components of ECG signals do not exceed 150 Hz, it is strategic to measure high frequencies to tune the models [27].

**Fig. 4:**
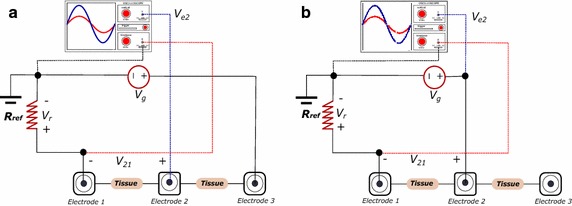
Setup for contact impedance measurement $(Z_{contact})$.** a** Circuit to calculate combined impedance $Z_{sum}$, it is equal to the sum of both, tissue impedance (skin impedance and subcutaneous tissue) and contact impedance due to electrode 2 ($Z_{contact}$).** b** Circuit to calculate impedance $Z_{23}$, it corresponds to sum of tissue impedance (skin impedance and subcutaneous tissue), contact impedance due to electrode 2, and contact impedance due to electrode 3

We used a variation of the method reported by Xie et al. [12]. The procedure involves determining the response of the electrodes when a sinusoidal voltage source is swept in frequency. This method is effective in measuring absolute magnitude of the impedance, however, does not allow the discrimination of the resistive and reactive components. To find such components individually, we performed the procedure based on the scheme in Fig. 4a. Instead of using a multimeter, we used a digital oscilloscope to estimate the magnitude and phase components of the contact impedance.

$$V_g$$ supplies AC signal ($$ 5~V_{pp} $$) to the circuit through the electrode 3; $$V_{e2}$$ corresponds to the voltage measured at electrode 2; the voltage $$V_r$$ at the reference resistance $$R_{ref}$$ is calculated with $$V_g$$ as the reference, and $$V_{21}$$ satisfies $$V_{e2} - V_r$$. $$V_r$$ and $$V_{e2}$$ are measured in the phasor form (magnitude and phase). The impedance is calculated as $$Z_{sum} = Z_{contact} + Z_{SB12}$$, where $$ Z_{contact}$$ represents contact impedance (skin/electrode) of a single electrode 1 and $$Z_{SB12}$$ represents the impedance of the subcutaneous tissue between the electrodes one and two. It can also be calculated by the expression:

1 $$\begin{aligned} Z_{sum}=Z_{contact}+Z_{SB12}=\frac{V_{21}}{I} \end{aligned}$$

where *I* is the current in the circuit and can be calculated as $I=\frac{V_r}{R_{ref}}$ (both values known). The circuit of Fig. 4b. allows determining the impedance $Z_{12}$, which satisfies $Z_{12}=2Z_{contact} + Z_{SB12}$. $V_g$ supplies AC signal ($5~V_{pp}$) to the circuit through the electrode 2; the voltage $V_r$ is measured at the reference resistance $R_{ref}$ and is obtained by using $V_{21}=V_g - V_r$. Thus, the impedance is calculated as:

2 $$\begin{aligned} Z_{12}=2Z_{contact}+Z_{SB12}=\frac{V_{21}}{I} \quad\text{where}\; I=\frac{V_r}{R_{ref}} \end{aligned}$$

Finally, contact impedance is calculated as $Z_{contact} = Z_{12}-Z_{sum}$.

#### Lissajous figures

Given an input signal *x*(*t*) and a phase-shifted output signal *y*(*t*) such as:

3 $$\begin{aligned} x(t)= \; & {} X_0sin(\omega t) \nonumber \\ y(t)= \; & {} Y_0sin(\omega t+\theta ) \end{aligned}$$

the Lissajous figure is generated when plotting $x(t)\ \text{vs}\ y(t)$ signals. The intersection of the figure with the *y* axis is identified and named $y_0$. The phase shift between the waveforms is calculated by the expression:

4 $$\begin{aligned} \theta =arcsin \left( \frac{y_0}{Y_0}\right) \end{aligned}$$

#### Correlation analysis

Cross-correlation is a measure of the similarity between two series as a function of one relative to the other. The cross correlation between a discrete input signal *x*(*n*) and an output signal *y*(*n*) is given by the expression:

5 $$\begin{aligned} r_{x,y}(l)=\sum _{n=-\infty }^{\infty }x(n)y(n-l) \end{aligned}$$

where *l* represents a shift in discrete time between signals. The aim is to find the value of *l* in such way that the maximum correlation between signals in obtained. From this value, the phase shift in degrees can be calculated from the equation:

6 $$\begin{aligned} \theta =\frac{360\cdot l\cdot F}{F_s} \end{aligned}$$

where *F* corresponds to frequency of the original wave and $F_s$ to the sample frequency from which the signals were acquired.

We determined voltages in the phasor form from their waveforms at the frequencies of interest. We designed a high-pass digital filter for eliminating the DC offset (Filter Designer App, Matlab, Mathworks, Inc.), with the following parameters: FIR, cut-off frequency = 0.05 Hz, order = 2000. Filter was applied off-line to both kind of signals (acquired from textile and reference electrodes), thus delays affected equally. The magnitude was calculated by obtaining the peaks of each wave. The phase was calculated using two techniques:

In order to compare the absolute impedance values of textile and disposable electrodes, we converted each data set of curves into a single scalar value. We have used the AUC score as a comparison parameter since the behavior of the magnitude of the impedance in the magnitude–frequency plots, decreases monotically with the increase of the frequency for all the samples. The use of the AUC score as a comparison criterion for spectral curves was used previously by Sarbaz et al. [28].

**Fig. 5:**
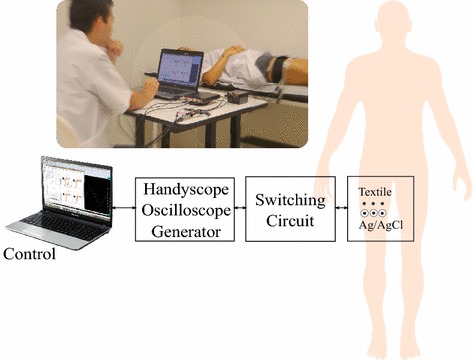
Contact impedance measurement scheme. Measurements are performed simultaneously in both textile and Ag/AgCl electrodes. The switching circuit has been used to interchange the measurement pins in the different configurations as explained before. Control is exerted automatically from a software application

We used a multifactorial ANOVA (either two-way or repeated variables) in cases where the statistical assumptions of normality and homoscedasticity were applicable. For those cases when the assumptions were not satisfied, we performed nonparametric tests, such as Kruskal–Wallis and Wilcoxon. The main factors evaluated were: the material of the textile electrode (cotton, cotton–polyester, lycra, silver-plated nylon, and polyester), and their behavior relative to the reference (textile electrode vs Ag/AgCl electrodes). The measurement scheme for this test is shown in Fig. 5.

We selected three different points of each leg to perform the measurements, to take into account the local variations of the skin. The tests are performed on the legs due to the ease of locating several electrodes for simultaneous measurements. In this way the probability of presence of motion artifacts and the interference from other bioelectric signals (such as ECG and breathing signals) is reduced.

### Polarization measurements

The electrode polarization is a consequence of an alteration of the charge distribution in the skin-electrode interface, and causes a baseline drift or DC potential/offset in ECG signals. Normally in practice, measuring ECG signals requires at least two electrodes connected differentially to an instrumentation amplifier that reduces the effects of common mode interference. ECG trace must be amplified and DC potential reduced. Electrochemical phenomena at the skin cause variations, as polarizations, on the skin-electrode interface, which results in interfering and modifying signals that are added to the desired ECG signal, although the characteristics of the electrodes are the same. Polarization at the skin-electrode interface was calculated by measuring a DC potential in open circuit at the terminals of a pair of electrodes attached to the skin. The potentials were registered once the patient remained one minute motionless to avoid instabilities in the skin-electrode interface, product of involuntary biomechanical movements. The skin-electrode interface is the largest source of interference due to polarization potentials. Polarization potentials are normally in the order of millivolts; however, when values exceed such order at the presence of action potential variations, the output of the amplifier is saturated, making the ECG signal difficult to extract and polarization potentials difficult to eliminate.

We set two independent acquisition channels (Cassy Lab, Label Didactics., Ltd.) to perform simultaneous measurements of DC potentials. The measurements were carried out both, in textile and reference electrodes in the same muscle group. We programmed a series of measurements by using four electrodes (two textiles electrodes and two Ag/AgCl electrodes), as reported by Rattfalt et. al. [19]. We used three different points of each leg, (sampling frequency = 10 sps), during approximately 30 min.

We calculated DC potentials for each type of electrode as the mean absolute difference between two consecutive samples. We used the standard average exchange ratio $\bar{X}_i$ suggested by Rattfalt [18, 19].

7 $$\begin{aligned} &\bar{X}_i=\frac{\sum _t|X_i(t)-X_i(t+1)|}{N-1} \quad t=0,1,2,...,N-1 \nonumber \\ &\bar{X}=\frac{\sum _i\bar{X}_i}{n} \end{aligned}$$

where $i$ denotes each particular individual; $n$ the total number of individuals for each electrode type, and $N$ the total number of samples. It is necessary to guarantee that the patient is motionless to avoid muscle signals product of involuntary movements.

**Fig. 6:**
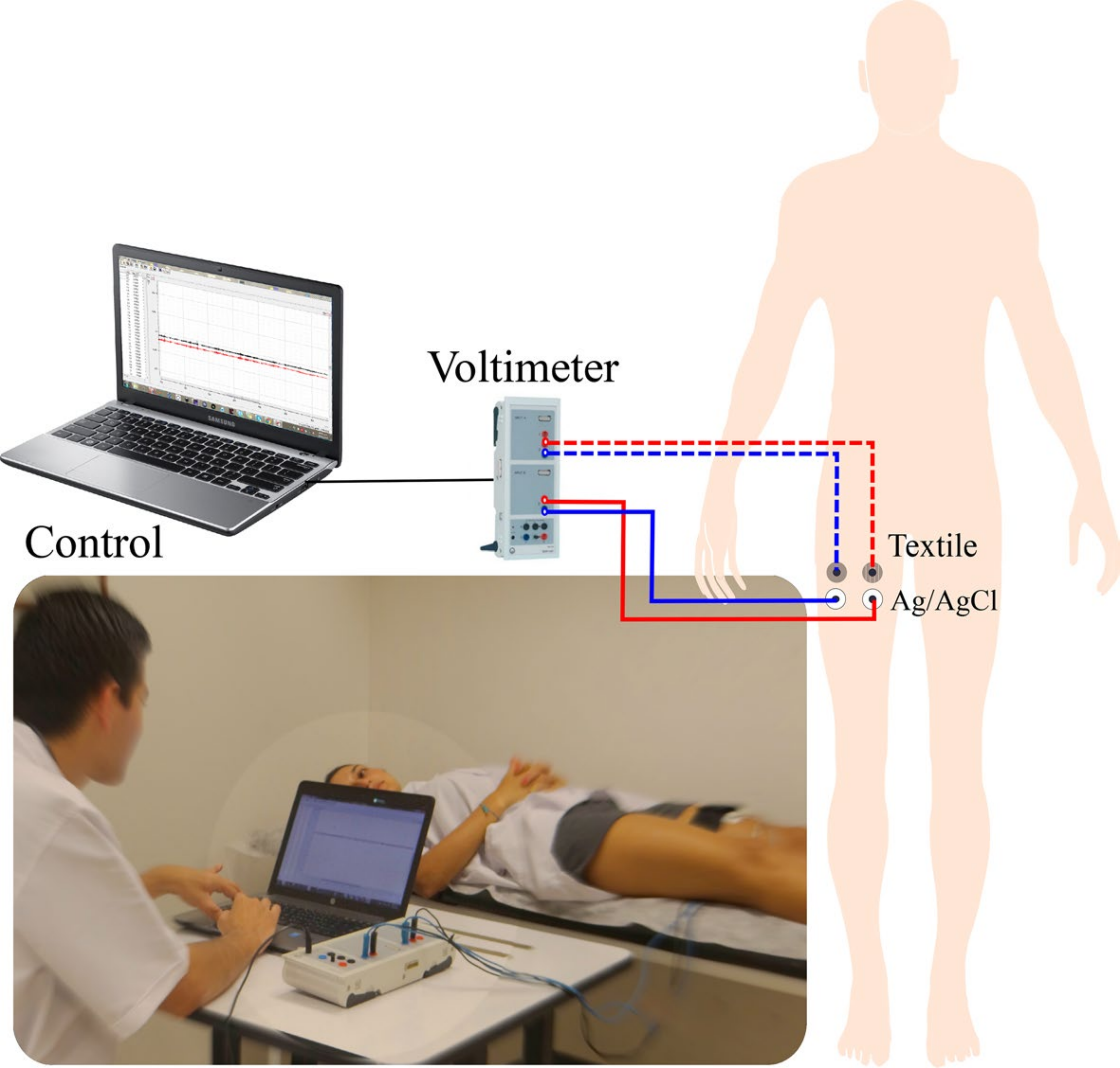
Open circuit polarization measurement scheme. Measurements were performed simultaneously in both textile and Ag/AgCl electrodes. We used a Cassy Lab sensor (Label Didactics., Ltd.) that allows to program and automatize a set of measurements from a software application at the PC

We used the interquartile range to eliminate the outliers in each set of observations. Each measurement series became a datum representing the average behavior of the electrode through the time. A two-way ANOVA analysis was used. We selected the type of electrode as the factor, the assumptions of normality (Kolmogorov–Smirnov,* p* = 0.2051) and homoscedasticity were satisfied (Levene test, *p* = 0.1149). The measurement scheme for this test is shown in Fig. 6.

### Noise measurements

**Fig. 7:**
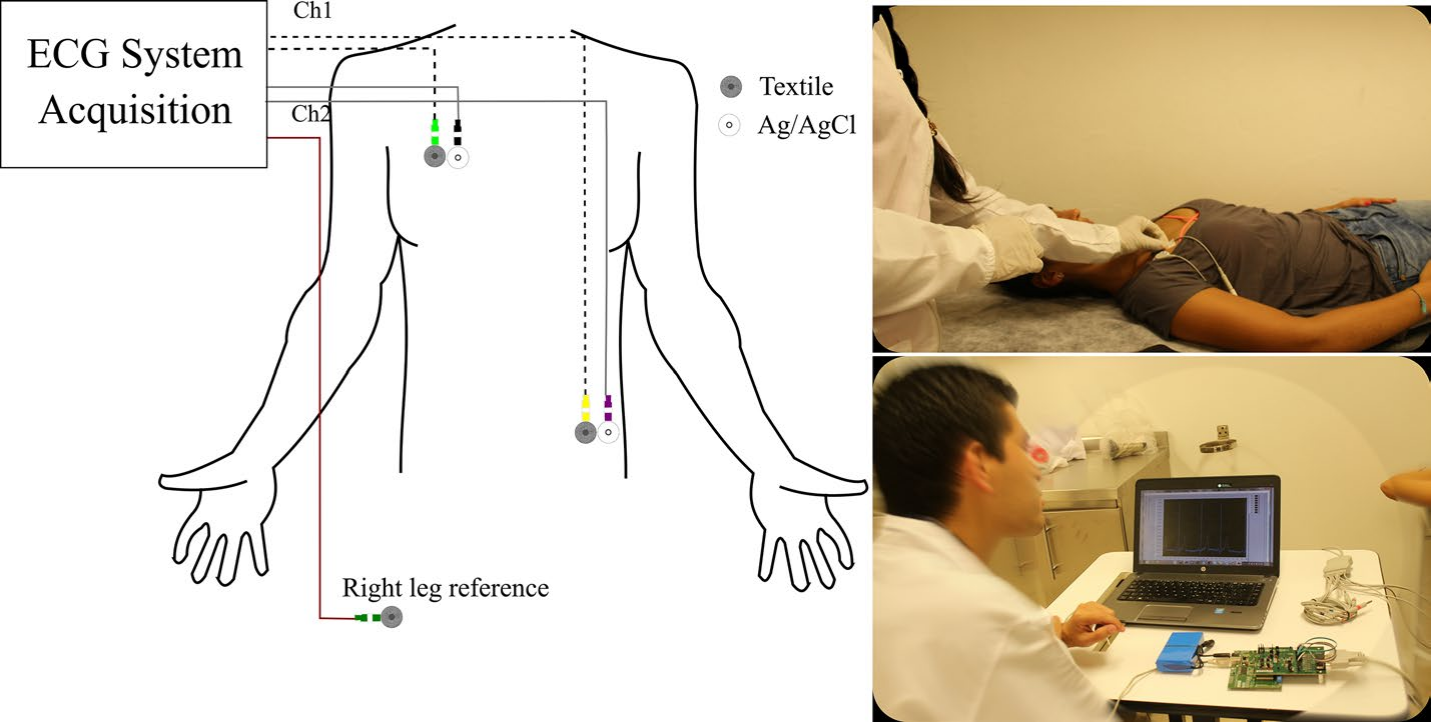
ECG signals measurement scheme. Measurements were performed simultaneously in both textile and Ag/AgCl electrodes. A circuit board based on the ADS1298 chip (Texas Instrument) was configured to acquire two channels at the same time

These experiments intended to quantify the noise level due to external interference, biological signals different to ECG, artifacts, and measuring equipment. We performed simultaneous measurements of the textile and commercial Ag/AgCl electrodes. The experiments consisted in capturing the same 1-lead ECG using different pairs of textile electrodes. We performed the measurements using lead II, as suggested by Takamatsu [29]. The acquisition process was conducted for a period of five minutes, where textile electrodes were attached to the skin by an elastic waistband. Figure 7 depicts the location of the electrodes and the connection to the electronics acquisition card EVM ADS1298 (Texas Instruments).

We designed a digital filter (Filter Designer App, Matlab, Mathworks, Inc.) for removing undesired components from the power supply and their corresponding harmonics (2 stopband filters, FIR filters, stop frequencies = 60 and 120 Hz respectively, windowing method = Kaiser, $\beta =0.5$, order = 150, broadband = 10 Hz) and attenuating the frequency components out of the range of cardiac signals (passband filter, FIR filter, band pass = 0.05–150 Hz, windowing method = Kaiser, $\beta =0.5$, order = 150), as is suggested in [30]. We performed three methods to analyze the data: noise power, cross-correlation coefficient, and segmentation.

Noise power quantifies the magnitude of the signal eliminated in the filtering process. The aim of this procedure is to identify which type of electrode has greater affectation by external interferences, biological noise, artifacts of muscle movement and breathing. The process involves determining the difference between the original and the filtered signal to calculate the average power.

8 $$\begin{aligned} &E=|ECG_{original}-ECG_{filtered}| \nonumber \\ &\bar{P}=\frac{1}{N}\sum _{i=0}^{N-1}E^2 \end{aligned}$$

where *E* is the absolute value resulting from the difference between the original and filtered signals. $\bar{P}$ is the noise power and *N* represents the number of samples.

The second method is the Pearson cross-correlation coefficient. Since the cardiac signals were recorded simultaneously with both, the textile and disposable electrodes, in the same area of the volunteer’s body, we expected two morphologically identical signals. However, they suffered a potential drift that was removed using a digital high pass filter described above. The normalized cross-correlation provides a value that expresses the similarity of two signals in terms of morphology; therefore, low values of the cross-correlation index suggest a large effect of noise on the ECG signals recorded by different electrodes.

The third process involves the use of a segmentation algorithm, which detects and quantifies complete P–Q–R–S–T waves. It uses the continuous wavelet transform, discrete wavelet transform, and Pan and Tompkins algorithm for the classification of the ECG signal, as reported by Bustamante et al. [31]. The error rate is calculated by dividing the number of complete ECG segments registered with the experimental material, against the number of ECG segments captured simultaneously with Ag/AgCl commercial electrodes.

### Long-term performance

The performance of the electrodes over time is affected by the wear of the material. We evaluated the degree of deterioration of the textile electrode quantifying its capacity to record complete ECG complexes that are morphologically similar to those recorded by Ag/AgCl electrodes. The signal acquisition process was the same as described in the noise measurement section. We evaluated each type of electrode (cotton, cotton–polyester, lycra, polyester and silver-plated nylon) for a period of 36 h on each of the four subjects. The volunteers continued with their daily lives but were asked to return to the laboratory to perform measurements spaced at 0, 1, 3, 7, 12, 24, 30 and 36 h. The measurements obtained at time 0 were added to the dataset for the noise analysis. During the entire process, we did not remove the textile electrodes from the patient’s skin; nevertheless, we adjusted them against displacements on each partial measurement. Due to the duration of the experiment, we replaced the disposable electrodes on each measurement. We did not performed additional measurements like contact impedance during these tests.

Signal processing (like filtering) was the same described in the noise measurements section. As this study focuses on the performance of fabrics, no especial filtering or higher order filter was needed. The selected range of frequencies permits components to pass through and provides a high-fidelity tracing for the P–Q–R–S–T ECG wave. Consequentially, we used the segmentation algorithm, introduced above, to extract the P–Q–R–S–T complex from the ECG trace and split it into single P–Q–R–S–T waves for individual analysis. Each ECG segment from the textile electrodes was compared with the segments, captured simultaneously, from the Ag/AgCl electrodes; then, the error rate is calculated by dividing the number of complete ECG segments registered with the experimental material, against the number of ECG segments captured simultaneously with Ag/AgCl commercial electrodes. We analyzed the data through a multivariate ANOVA of repeated variables.

## Results

According to the data input given by Mestrovic (2016), the calculated conductivity value was 2.64 S/cm for the disposable commercial Ag/AgCl 3M electrodes used in this study. Two orders above from cotton–polyester and lycra: 337 and 393 mS/cm respectively [32]. It is clearly seen that lycra performs sensibly better than cotton–polyester as an evidence of the affinity of both materials to the PEDOT:PSS solution. The order of the conductivity values demonstrate that the performance of lycra is actually better that cotton–polyester to enhance ionic transfer through the skin-electrode interface. Disposable electrodes have the highest conductivity among the samples, including those reported in Pani et al. Conductivity data is validated by Fig. 12b; however, data reported by Pani does not correspond to impedance analysis. It is clearly visible that lycra and cotton–polyester electrodes have a similar performance. Conductivity of Med-Tex P130 was not calculated as it is not an isotropic material, which means it does not have uniformed conductivity, due to it is plated for silver ionic release; in fact, we presume the dispersion of the electrodes made Med-Tex P130 is high.

**Fig. 8:**
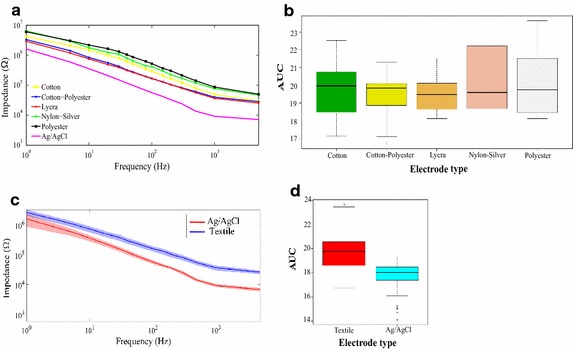
Contact impedance. **a** Average of the magnitude of the contact impedance of each material versus frequency. Each of the lines of the graph represents the average value of the magnitude of the contact impedance evaluated on the different test subjects. **b** Box and whisker plot with the value of the area under the curve (AUC) of each of the materials. The AUC is a numerical value that represents the value of the area under the curve of the impedance spectral signals. **c** Shadow plot of the contact impedance magnitude versus frequency: the solid line represents the average value, the shadow is the standard deviation of the values around the average. Textiles (blue), Ag/AgCl (red). **d** Box and whisker plot with the value of the area under the curve comparing textile versus Ag/AgCl electrodes

Figure 3 shows the characteristics of the weaves of each fabric used in the study. Cotton and cotton–polyester exhibit a similar pattern, the fabric is made with thick yarns (approximately 200 µm) intertwined at short intervals. Only a few empty spaces between yarns are appreciable. Despite their physical similarities, cotton–polyester is closer to lycra in terms of the contact impedance than cotton. Lycra presents thinner yarns than cotton (150 µm approximately), the pattern of the fabric is linear, the yarns are nearby and tight, which leaves very few empty spaces. Indeed, lycra exhibit the lowest impedance values. Nylon–silver exhibits a pattern similar to lycra, its network of intertwined yarns leaves few empty spaces; however, its contact impedance is higher. Polyester presents the thinnest yarns (approximately 100 µm); besides, they are very far apart, which causes many empty spaces. It may explain its poor performance in establishing a good skin electrode interface, which causes the highest contact impedance values (the black line in Fig. 8).

We recorded and plotted the data from the four experiments separately for each individual. We assessed the electrical performance of textile electrodes by analyzing impedance magnitude, polarization variability, ECG morphology deviations, and proneness to electrical noise. We used statistical tools to compare textile electrodes against Ag/AgCl commercial electrodes.

### Contact impedance measurements

Figure 8a shows a Bode plot of the average impedance magnitude at the selected frequency range, from 0.1 to 10^4^ Hz. The statistical analysis of the data showed that the assumptions of normality and homoscedasticity were not satisfied; thus, we used the Kruskal–Wallis test to interpret the data. Figure 8b presents a box plot with the distribution of the data. The figure was constructed from data taken from four test subjects at six test points, corresponding to 24 measurements per material simultaneously with the reference electrodes. The data were analyzed using a non-parametric Kruskal–Wallis test yielding a *p* = 0.8684, *confidence* = 95% indicating that there are no significant differences between electrode types. Figure 8c shows a shadow plot to represent the impedance average of textile materials (blue) compared to the reference Ag/AgCl electrodes (red). Shadow represents the standard deviation. Thus, it is possible to appreciate a clear difference in the impedance magnitude of the two groups. Figure 8d presents a comparison of the data distribution by groups In total, 24 samples per material (120 measurements) and the same number of measurements with the reference electrodes were used. In this case, the assumptions of normality and homoscedasticity were not satisfied either; hence, it was necessary to perform a Wilcoxon test. The test indicated significant differences between treatments (*p* = 2.2 × 10^–16^,* confidence* = 95% ) where the textile electrodes, generally, presented higher contact impedances compared to Ag/AgCl commercial electrodes; besides, they had higher dispersion.

### Polarization measurements

We obtained minimal variability in polarization potentials along the measurements (we only noticed small changes due to muscular activation). The average polarization level was 15.4 mV, the standard average exchange ratio calculated between consecutive measures was 269.29 µV every 0.1 s. Under the same conditions, Ag/AgCl electrodes show an average polarization level of 2.54 mV (standard average exchange ratio = 163.56 µV every 0.1 s). Detailed results of this test are presented in the Table 1.

**Table 1:** Measurements of the average polarization potential and of the variability between consecutive samples taken at time intervals of 0.1 s registered on each of the four test subjects, with each type of textile material (bold values) compared with commercial electrodes of *Ag*/*Agcl* (cursive values)

Subject	Measurements of average polarization potential with each type of electrode in mV				
	Cotton	Cotton–polyester	Lycra	Silver-plated nylon	Polyester
Female 1	**11.26**	**6.27**	**13.96**	**10.70**	**9.04**
	*1.93*	*1.25*	*0.73*	*1.93*	*1.31*
Female 2	**24.57**	**8.04**	**15.12**	**12.61**	**22.24**
	*1.13*	*1.13*	*1.32*	*1.32*	*1.13*
Male 1	**7.61**	**56.8**	**3.20**	**30.16**	**5.08**
	*1.02*	*4.50*	*1.02*	*4.50*	*1.02*
Male 2	**12.36**	**31.12**	**11.80**	**2.75**	**10.71**
	*5.08*	*6.47*	*5.07*	*5.07*	*6.47*

Subject	Measures of the variability of the average potential with each type of electrode in µV				
	Cotton	Cotton–polyester	Lycra	Silver-plated nylon	Polyester
Female 1	**667.14**	**353.02**	**401.56**	**712.9**	**406.84**
	*589.16*	*364.56*	*393.81*	*589.13*	*349.61*
Female 2	**133.87**	**325.02**	**259.18**	**282.46**	**236.30**
	*103.84*	*100.55*	*91.11*	*91.23*	*103.78*
Male 1	**399.55**	**164.78**	**393.14**	**108.74**	**428.69**
	*121.75*	*68.55*	*121.29*	*61.83*	*122.21*
Male 2	**35.17**	**43.33**	**27.08**	**29.41**	**34.01**
	*21.02*	*22.72*	*22.47*	*19.97*	*20.51*

**Fig. 9:**
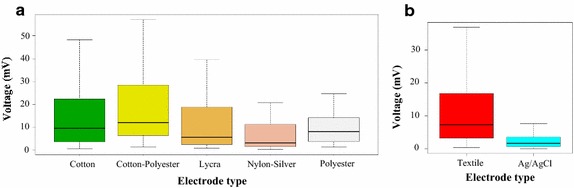
Average polarization potential drift for different materials and series of measurements. **a** Box and whisker plot of the average polarization drift of different materials. **b** Box and whisker plot of the average polarization drift comparing textile vs Ag/AgCl electrodes

Silver-plated nylon electrodes showed the lowest polarization potential value (*p* = 0.004035). Figure 9a depicts the average behavior along the time; results do not evidence significant differences among the other materials. Figure 9b presents a general comparison of the electrical behavior of textile and Ag/AgCl electrodes. Data did not meet the normality assumption, therefore we performed a Wilcoxon test. It confirmed that electrodes treated with PEDOT:PSS have higher polarization compared to Ag/AgCl.

### Noise measurements

**Fig. 10:**
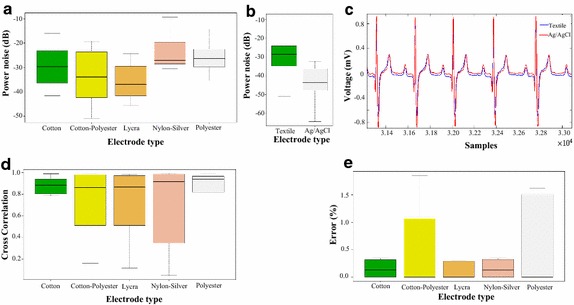
Noise measurements. **a** Box and whisker plot of the average noise power quantified from a filtering process.** b** Box and whisker plot of the average noise power comparing textile vs Ag/AgCl electrodes.** c** Plot of the average Pearson cross-correlation coefficient calculated between ECG signals acquired simultaneously (textile vs Ag/AgCl).** d** Segment of an ECG signal simultaneously recorded with textile (blue) and Ag/AgCl (red) electrodes.** e** Average percentage of error in the detection of ECG signal segments.

Statistical analysis showed significant differences in the behavior of the materials ($p=0.01945$), particularly that the lycra electrodes are less sensitive to noise than silver-plated nylon electrodes, as depicted in Fig. 10a.

Additionally, we compared the overall performance of the textile electrodes against the reference electrodes Ag/AgCl. For this purpose we performed a two-way ANOVA analysis using as factor the type of electrode (textile or disposable), see Fig. 10b. Assumptions of normality (Shapiro–Wilk* p* =  0.09372) and homoscedasticity (Levene* p* = 0.2317) were satisfied. We observed that the disposable electrodes present a significantly lower noise levels compared with textile electrodes (*p* = 9.17 × 10^–11^).

Cross-correlation analysis depicted in Fig. 10d quantifies the similitudes between the signals recorded with the electrodes under test (Fig. 10c). In general, the correlation values are higher than 80%. Besides, we observed no statistically significant differences between textile electrodes performance.

The ability of the electrodes to acquire ECG signals was tested by the segmentation algorithm. The error rate was determined and tabulated in Table 2 based on a Kruskal–Wallis test, complementary results of comparisons are presented on Fig. 10e. Such test showed no significant differences between different types of treatments (*p* = 0.9965). In Table 2, 92.5% of the measurements, yield that the percentage error was less than 2%, such values are within the tolerance of the algorithm (98%).

**Table 2:** Number of subjects in the error range versus electrode type

Type of electrode	Number of subjects			
	Error range			
	0%	0–2%	2–5%	> 5 %
Cotton	4	4	0	0
Cotton–polyester	5	2	0	1
Lycra	5	2	1	0
Sylver-plated nylon	4	4	0	0
Polyester	5	2	0	1

The error is calculated by counting the number of complete ECG signals in a time interval of 5 min using the segmentation algorithm. Then, number of subjects within the percentage errors: 0, 0–2, 2–5% and higher than 5% were quantified

### Long-term performance

**Fig. 11:**
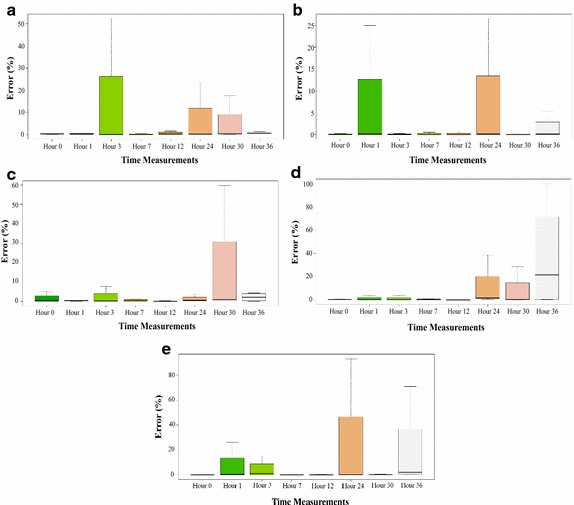
Long-term performance: segmentation error percentage versus time. Each one of the box diagrams represents the percentage of error in the determination of ECG signals of the textile electrodes with respect to the commercial electrodes, in the different measurement time periods. Most of the graphs show that the median of the measurements is below 5%.** a** Cotton electrodes.** b** Cotton–polyester electrodes.** c** Lycra electrodes.** d** Nylon–silver electrodes.** e** Polyester electrodes.

We performed eight measurements, lasting 5 min each, in a time interval of 36 h, we counted the number of complete P–Q–R–S waves using the segmentation algorithm. Figure 11 shows the results of each type of material.

There is no evidence to establish differences in the number of complete ECG signals detected during the test. In general, after 36 h, the quality of the captured ECG signals is similar to the obtained at 0 h. Except for the very last measurement with silver-plated nylon electrodes, in all cases, the average percentage error was less than 5%. Silver-plated nylon electrodes are the only ones that presented an apparent relationship between the increase in the percentage error and time. In Fig. 11 the results are graphically presented for each type of material.

**Fig. 12:**
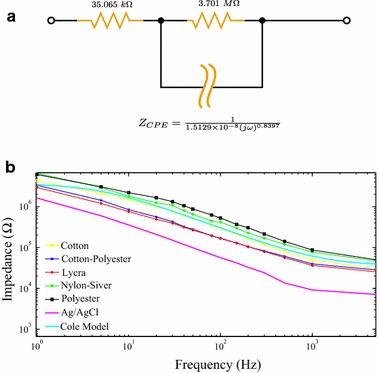
Circuit model of contact impedance for textile electrodes.** a** Single dispersion Cole impedance model.** b** Continuous cyan line fits the model, other lines are the values of impedance magnitude obtained in the research

We propose a single dispersion Cole impedance model for textile electrodes treated with PEDOT:PSS (Fig. 12). The impedance parameters obtained for this model were $R_\infty =35.065\;\text{k}\Omega$, $R_1=3.701\;\text{M}\Omega$, $C_1=15.129\;\text{nF}$ and $\alpha _1=0.8397$. This static model only intends to represent a simplification of the data acquired in this study. The model can be adjusted by increasing the number of tests and individuals; thus, the model can be tuned to the specific set of data. The high variability of biological systems increases the difficulty of obtaining deterministic and predictive models. Therefore, reference models become a valid alternative for preliminary examinations.

The main results of the research are summarized in Table 3.

**Table 3:** Summary of the main findings of the investigation

Parameter	Textile electrodes results	Textile electrodes vs Ag/AgCl electrodes
Contact impedance measurements	There are not significant differences between the different textile electrodes. The impedance is resistive-capacitive type reflected in the decrease in impedance with the increase in frequency	There are significant differences between textile electrodes and commercial electrodes. The contact impedance in the Ag/AgCl electrodes is lower
Polarization measurements	There are not significant differences between textile electrodes. The polarization signals are that they do not present variation in time, that is, the signals tend to be constant, which implies that they can be easily removed using analog instrumentation (AC coupling circuit) of by digital filtering (DC coupling). This is the most important finding in polarization measurements	There are significant differences between textile electrodes and commercial electrodes. The contact impedance at the electrodes of Ag/AgCl is lower. The average polarization obtained with the textile electrodes is 15.4 mV while the average polarization obtained with the commercial electrodes is less than 2 mV
Noise measurements	There are not significant differences between textile electrodes. In spite of the high measured polarization, textile electrodes correctly acquire cardiac signals with all their morphological components	The noise signals measured with textile electrodes are greater than with commercial electrodes; however, this noise is easily removable using digital filtering and the morphological components of the ECG signal can be identified successfully
Long-term performance measurements	After 36 h of adhering the electrodes to the test subject, the textile electrodes remain functional in the acquisition of cardiac signals with all their components. The only electrode that shows deterioration for the acquisition of the ECG signal are the nylon–silver electrodes	No tests were performed with commercial electrodes in long-term. In each measurement new Ag/AgCl electrodes are used to compare with the textile electrodes. The Ag/AgCl electrodes used are disposable. According to hospital standards, disposable electrodes should be replaced at least every 24 h and after a long time can generate irritation on the skin

## Discussion

ECG signals were correctly registered by a set of textile electrodes treated with a conductive polymer (PEDOT:PSS). Materials under test were cotton, cotton–polyester (65% cotton, 35% colyester), lycra, polyester, and MEDTEX P-130. No gel, substrate or adhesive material was used to improve the ionic conductivity. Experiments confirmed that these materials can be used in the fabrication of wearable sensors of daily use. Materials tested are suitable for applications where the use of disposable electrodes is not practical.

Fabrics in Fig. 3 contain fibers with a coating of PEDOT:PSS with no visible deterioration at microscopic level. Due to previous conductivity calculations, we determined that the combination of highly conductive solution of PEDOT:PSS with cotton, lycra, cotton–polyester, and polyester provides an acceptable surface resistance for medical applications, specially monitoring of ionic transfers. All conductivity-related properties are connected with its fibrous structure. Resistance is associated with contact resistance between neighboring yarns and numerous contact points at the crossing between yarns in each fabric [33]. Figure 3b, c show that the manner in which the yarns of lycra and cotton–polyester are arranged on the surface of the fabrics does not impact significantly their resistance, which was observed in the impedance responses. However, when they are compared to Fig. 3d, the impedance differs notably; indeed, the gaps between each yarn represent an increment on the impedance as a consequence of the material resistance. From the observations to the fibers, we identified that the anisotropy may result from the different number of warp and weft yarns per length unit. It is visible that the current does not spread in uniformly on the surface. As we did not care about the orientation of the surface interface from the fabrics, we cannot address any affirmations about the direction of current flows along the yarns and the detailed effects of the gaps between them. However, the arrangement of the yarns, which was multi-directional, show better results in the impedance analysis and conductivity calculations, we presume that the interlacing yarns conducts to higher current captures, caused by the isotropy of electroconductive properties of fabrics shown in Fig. 3b, c. Nylon–silver and polyester showed worst performance doe to their anisotropic structure.

We determined that materials treated with PEDOT:PSS presented no statistically significant differences in acquiring ECG signals. MEDTEX P-130 based electrodes only presented a better performance in polarization tests; hence, showed a slight tendency to have poorer performance in ECG signal acquisition. Lycra based textile electrodes exhibited a highly reliable behavior, represented in lower impedance mean values and lower dispersion in the different repetitions.

Notwithstanding the multiple factors that influence the skin impedance, and the high variability present in contact impedance data, even in the same individual, we confirmed that the contact impedance is higher in textile electrodes compared to commercial electrodes. One of the factors that could strongly influence these results was the effective area of textile electrodes (4 cm^2^), an analysis that was out of the scope of this work. Effective electrode area affects the skin-electrode interface and its impedance which actively influences the acquired ECG signal. In fact, the relationship between the effective area of the electrode and contact impedance was studied by Puurtinen et al. [20]. It is also well known that high contact impedance is balanced with high and ultra-high input impedance electronic systems [34, 35].

It is important to highlight that all the tests reported in this paper were performed with dry electrodes. We did not use any type of gel or electrolyte that help to improve the conductivity of the skin-electrode interface. This could explain the high values of contact impedance with respect to the Ag/AgCl electrodes. Actual applications where electrodes are incorporated to acquire bioelectric signals are intended to be into the wardrobe in a natural way for the end user. Wearable devices should not become an invasive element, difficult to use and manipulate.

This work aimed to elucidate which material provides advantages for the manufacture of garments that allow the continuous monitoring of electrocardiographic signals. However, since there is no significant difference between the electrical characteristics of the materials, it is relevant to conduct studies focused on the mechanical properties of textile materials. Besides, strategies should be sought to improve the fitting of the electrode with the skin, in order to improve the effective area of contact.

Previous work demonstrated that textile electrodes have a significant effect on charge transfer. This is due to the complex contact area created by the woven structure, and since the surface of the electrode is not completely parallel to the skin. Many variables inherent to textile materials contribute to the high variability of the contact impedance, for instance, the number of fibers per cross-section, fiber properties, conductive polymer adhesion to the fibers, fiber density, and hairiness [36]. Figure 3 shows the physical appearance of the surface of the different fabrics used in this study. The woven structures apparently influence the contact impedance through the contact area formed with the skin. Polyester shows the highest impedance value, which may correspond to the empty spaces that remain between the fibers. On the other hand, lycra has the lowest impedance values, which may obey a better contact area, created by a better disposition of fibers tightly arranged.

Textile electrodes treated with PEDOT:PSS have a higher extent of polarization level than conventional ones, and the MEDTEX P-130 based electrodes (mean value: 15.4 mV). The results did not show substantial changes under conditions of complete rest; however, were slightly affected by artifacts generated by muscle activation. The potential polarization effect tends to become uniform over time (variability < 0.3 mV every 0.1 s), which contributes to its elimination through the use of analog electronic circuits.

On the segmentation process, the error rates were generally under 2%. Signals taken with textile electrodes showed a greater presence of noise than (Ag/AgCl) commercial electrodes. Nonetheless, all the segments of the ECG signal were identified properly. MEDTEX P-130 electrodes are more sensitive to noise than the other textile electrodes.

Long-term performance measurements showed that after 36 h the electrodes treated with PEDOT:PSS continue to have an adequate performance, i.e. ECG signals were clearly identified. MEDTEX P-130 based electrodes showed deterioration of the ECG signal during the test, likely as a consequence of the interaction with biological fluids. There is no evidence that relates changes of the properties of the electrodes over the time; however, misreadings can be attributed to a poor contact to the skin as a result of a movement, displacement of the material or momentary disconnection during the test. Due to the duration of the test, data was only visualized off-line, after the segmentation process. The duration of the long-term performance measurements was conditioned by the availability of laboratories and the volunteers. Takamatsu et. al [29] reported reliable results after 72 h using textile electrodes with similar features.

We propose, as future work, the development of monitoring systems using wearable sensors that incorporate PEDOT:PSS treated electrodes. Investigations should focus on the behavior of textile electrodes for long periods of time, material behavior after washing processes, noise and artifacts during physical activity, and the effects of sweating on the quality of the ECG. Bearing in mind that the material is intended to be used in wearable devices, a future study is necessary that contemplates the use of dynamic tests. It is also important how the effective area, the morphology, thickness of the polymer on the fabric and other characteristics can modify the contact impedance of the skin electrodes and their effect on the quality of the acquired ECG signals.

## Conclusions

Contact impedance, polarization, and noise level tests showed significant differences in favor of Ag/AgCl commercial electrodes. We found that fabrics treated with PEDOT:PSS such as cotton–polyester (65% cotton, 35% polyester), lycra and polyester are suitable for activities that do not involve diagnosis. Although, they have a considerable advantage over disposable electrodes, which must be replaced at least every 24 h.

Long-term performance tests demonstrated that fabrics treated with PEDOT: PSS are functional after 36 h of continued use. They allowed to acquire ECG signals as at the beginning of the test; however, electrodes constructed with silver-plated nylon showed considerable deterioration of the ECG signal after 24 h.

We measured noise level under 5% on fabrics treated with PEDOT:PSS and silver-plated nylon. Such fabrics could be used as primary sensing elements of an ECG monitoring system.

The average polarization level measured on the textiles under test was 15.4 mV. Polarization levels were constant and only affected by involuntary muscle movements performed by the individuals. Such values can be removed by DC coupling mechanisms or by digital filtering. Silver-plated nylon electrodes showed superior performance than electrodes treated with PEDOT:PSS, in comparison to the behavior of Ag/AgCl electrodes.

None of the tests yielded statistically significant evidence that permits to determine that one PEDOT:PSS treated material used in textile electrodes has a superior performance compared to others. Therefore in the development of wearable ECG signals acquisition systems, the type of material is not an aspect to be considered from the point of view of signal quality and electrical behavior. Future studies should focus on mechanical characterization of the material to obtain an adequate coupling to the skin to orientate the applications to the development of systems for athletes, people in rehabilitation or at risk of heart disease or prevention systems, generation of early warnings and promotion of self-care.
